# Cytochrome P450 monooxygenase/soluble epoxide hydrolase-mediated eicosanoid pathway in colorectal cancer and obesity-associated colorectal cancer

**DOI:** 10.18632/oncoscience.488

**Published:** 2019-08-23

**Authors:** Jianan Zhang, Katherine Z. Sanidad, Guodong Zhang

**Affiliations:** ^1^ Department of Food Science, University of Massachusetts, Amherst, MA, USA; ^2^ Molecular and Cellular Biology Program, University of Massachusetts, Amherst, MA, USA

**Keywords:** Eicosanoids, cytochrome P450 monooxygenase, soluble epoxide hydrolase, colorectal cance

## Abstract

Colorectal cancer (CRC) is the third most common cancer and the second leading cause of cancer-related deaths in the United States. Furthermore, it is well established that obese individuals have high risks of developing CRC, and obesity-associated CRC represents an unmet medical problem in the United States. Using a metabolomics approach, our recent research supports that the cytochrome P450 (CYP) monooxygenase/soluble epoxide hydrolase (sEH)-mediated eicosanoid pathway could play critical roles in the pathogenesis of CRC and obesity-associated CRC. Here in this review, we discuss recent studies about the roles of the CYP/sEH eicosanoid pathway in the pathogenesis of these diseases.

## INTRODUCTION

In the United States, there are ~130,000 new cases and ~50,000 deaths caused by colorectal cancer (CRC) every year, making CRC a serious health problem [1]. It is important to identify novel therapeutic targets of CRC in order to develop safe and effective approaches for prevention and/or treatment. Eicosanoids, which are metabolites of polyunsaturated fatty acids (PUFAs) produced by cyclooxygenase (COX), lipoxygenase (LOX), and cytochrome P450 (CYP) enzymes, are endogenous lipid signaling molecules involved in the regulation of inflammation and hemostasis [2]. Substantial studies have shown that the COX and LOX pathways play critical roles in the pathogenesis of CRC, and these two pathways have been targeted for therapeutics of CRC [3, 4]. Notably, human clinical trials support that COX inhibitors, including non-steroidal anti-inflammatory drugs (NSAIDs) and COX-2 selective inhibitors (coxibs), are among the most effective agents to reduce risk for CRC [5, 6]. In fact, the FDA has approved celecoxib (Celebrex®, a coxib) to prevent precancerous colorectal polyps. However, long-term high-dose use of NSAIDs or coxibs can cause life-threatening adverse effects, jeopardizing their clinical applications [7]. To date, the roles of the CYP pathway (the third branch of the eicosanoid signaling pathway) in the pathogenesis of CRC are largely unknown [2].

### Roles of the CYP monooxygenase pathway in CRC

The metabolism of PUFAs by CYP monooxygenases (largely the CYP2C and CYP2J isoforms) leads to the formation of epoxygenated fatty acids (EpFAs) [8, 9]. EpFAs, notably epoxyeicosatrienoic acids (EETs) derived from arachidonic acid (ARA, 20:4ω-6), are important lipid signaling molecules involved in the regulation of many important biological processes such as inflammation and vascular tone [2]. EpFAs are metabolically unstable and have a half-life of several seconds *in vivo*, mainly because they can be rapidly degraded by soluble epoxide hydrolase (sEH) [2]. Currently, pharmacological inhibitors of sEH, which stabilize EpFAs *in vivo*, are being evaluated in multiple human clinical trials [10, 11].

Our recent research supports that the CYP monooxygenase pathway could play an important role in regulating the development of CRC [12]. First, we found that the CYP monooxygenase pathway was upregulated in CRC. Notably, the concentrations of EpFAs were significantly increased in both the plasma and colons of azoxymethane (AOM)/dextran sodium sulfate (DSS)-induced CRC mice compared with control healthy mice. Furthermore, the expressions of CYP monooxygenases were increased in the colon tissues of AOM/DSS-induced CRC mice, as well as human CRC cells. Second, we found that CYP monooxygenases contributed to colon tumorigenesis, since pharmacological inhibition or genetic ablation of CYP monooxygenases suppressed AOM/DSS-induced colon tumorigenesis in mice. Third, we discovered that the pro-CRC effects of CYP monooxygenases were, at least in part, mediated by epoxyoctadecenoic acids (EpOMEs), which are metabolites of linoleic acid (LA, 18:2ω-6) produced by CYP monooxygenases. Indeed, we found that treatment with EpOMEs, at nM concentrations, increased inflammation in RAW 264.7 mouse macrophage cells and HCT-116 human colon cancer cells. In addition, continuous infusion with low-dose EpOMEs exaggerated AOM/DSS-induced colon tumorigenesis in mice. Our results are largely in agreement with previous research, which showed that CYP monooxygenases promoted tumor growth and metastasis of other types of cancers in xenograft tumor models [13–16]. Together, these results support that the CYP/EpOME axis could contribute to the pathogenesis of CRC.

Our results support that CYP monooxygenases could be potential therapeutic targets of CRC, since we showed that the inhibition or deletion of CYP monooxygenases suppresses colon tumorigenesis in mice [12]. Recent research supports that some CYP enzymes, notably CYP1B1, are overexpressed in tumor cells and could be promising therapeutic targets for cancer treatment [17]. Pharmacological inhibitors or gene therapy targeting CYP enzymes are being evaluated as anti-cancer therapeutics [18]. Based on our study, it is feasible to also target CYP2C and CYP2J monooxygenases for CRC prevention and/or treatment. Some previous studies have screened libraries of FDA-approved drugs and found that some of these compounds, such as telmisartan (Micardis®, an anti-hypertensive drug) and gemfibrozil (Lopid®, a lipid-lowering drug), are potent inhibitors of the CYP2C/2J enzymes [19, 20, 21]. Results from these studies suggest that telmisartan and gemfibrozil, as well as other FDA-approved drugs, could be repurposed as therapeutics for CRC. It would be important to test whether these CYP inhibitors could inhibit CRC in pre-clinical models, thus facilitating potential clinical trials to test whether these FDA-approved drugs could be repurposed for preventing and/or treating CRC. However, we should note that a complete inhibition of these enzymes might cause adverse effects, since many CYP monooxygenases play critical roles in some critical biological actions [8]. Indeed, our study showed that compared with wild-type (*Cyp2c*^+/+^) mice, heterozygous (*Cyp2c*^+/−^) mice showed no observable adverse phenotypes at basal conditions and also reduced development of AOM/DSS-induced colon tumorigenesis; however, the homozygous (*Cyp2c*^−/−^) mice showed severe liver inflammation at basal conditions (without AOM/DSS treatment) and quickly died upon AOM/DSS stimulation [12]. These findings suggest that complete inhibition of CYP monooxygenase could disrupt some critical biological processes, which remain to be elucidated, resulting in adverse effects. However, these adverse effects could be mitigated by adjusting the doses of CYP monooxygenase inhibitors in cancer therapy, or developing highly selective inhibitors targeting specific CYP monooxygenase isoform(s).

Our finding supports EpOMEs, which are LA metabolites produced by CYP monooxygenases, as important endogenous regulators of CRC [12]. We found that treatment with EpOMEs, at low doses, induces inflammation *in vitro* and exaggerates colon tumorigenesis *in vivo* [12]. Our findings are largely in agreement with previous studies, which showed that EpOMEs have a series of detrimental effects, such as inducing chemotaxis, inflammation, cardiovascular diseases, and pulmonary injury [22–27]. In human studies, EpOMEs are associated with multiple organ failure and adult respiratory distress syndrome in severe burn patients, and are termed “leukotoxins” [23, 25, 28]. EpOMEs could be further metabolized by sEH to form the corresponding fatty acid diols dihydroxyoctadecenoic acids (DiHOMEs) [2]. Similar to EpOMEs, DiHOMEs have also been shown to induce chemotaxis, tissue injury, and cause mortality in animal models [28, 29]. Together, these results show that EpOMEs, as well as their downstream metabolites, could contribute to the pathogenesis of many human disorders, including CRC.

A better understanding of the molecular mechanisms of EpOMEs could help to develop novel strategies for CRC treatment. Many eicosanoids, such as COX-produced prostaglandin E2 (PGE2) and LOX-produced leukotriene B4 (LTB4), act by binding to G-protein coupled receptors (GPCRs) [30]. Emerging research supports that CYP monooxygenase metabolites, notably EETs, also act via receptor-dependent mechanisms. Recent studies have shown that EETs bind to cell membrane-bound proteins in a high-affinity, specific, and saturable manner, supporting the presence of potential receptor(s). Using a synthetic photo-affinity probe, Chen *et al*. suggested a potential high-affinity EET binding protein in U937 cells and vascular cells [31]. In addition, Ding *et al*. showed that downregulation of G-proteins abolished the biological action of EET *in vitro*, supporting that the bioactivity of EET is GPCR-dependent [32]. Further *in vitro* cell studies by Park *et al*. showed that the increase of MAP kinase (MAPK)-mediated ERK phosphorylation by 11,12-EET were inhibited by using a specific GPR40 antagonist or siRNA-mediated GPR40 silencing, indicating that GPR40 was a low-affinity EET receptor in vascular cells and arteries [33]. Together, these results support that similarly to other eicosanoids, the CYP monooxygenase metabolites could also exert their biological actions via binding to specific cellular targets or receptors. To date, the specific receptors or direct cellular targets of EpOMEs are unknown, hampering our understanding of the molecular mechanisms of EpOMEs. It would be important to elucidate the direct cellular targets or receptors of EpOMEs, since the identified proteins could serve as novel therapeutic targets of CRC, and potentially other human disorders.

Based on the potent and broad-spectrum adverse effects of EpOMEs, antagonists of EpOMEs could be developed and used as novel therapeutics. Previous research supports that it is feasible to develop antagonists of CYP monooxygenase metabolites, though their receptors remain unknown. Gauthier *et al*. designed and developed 14,15-epoxyeicosa-5-enoic acid (14,15-EEZE) as a potential antagonist of 14,15-EET [34]. These two compounds have been shown to exert opposite actions: Panigrahy *et al*. showed that 14,15-EET enhanced tumor growth and metastasis in mice, while 14,15-EEZE inhibited tumor growth and increased mouse survival [14]. In addition, recent research showed that it is also feasible to develop synthetic mimics of CYP monooxygenase metabolites as novel therapeutics. Previous research showed that 17,18-epoxyeicosatetraenoic acid (17,18-EEQ), an eicosapentaenoic acid (EPA, 20:5ω-3) metabolite produced by CYP monooxygenases, has potent cardioprotective effects [35], and metabolically stable EEQ analogs were developed as potential treatments of cardiovascular and inflammatory diseases [36]. Together, these findings support that CYP monooxygenase metabolites, including EpOMEs, are important structural targets to develop stable antagonists or mimics as novel therapeutics.

### Roles of the CYP eicosanoid pathway in obesity-associated CRC

More than one-third of adults in the United States (~78.6 million) are obese [37], and obese individuals have a 30-60% higher risk of developing CRC compared with non-obese individuals [38, 39]. Considering the obesity epidemic and potential lethal consequence of CRC, obesity-associated CRC is a serious health problem in the United States. However, the mechanism by which obesity increases the risk for CRC is not well understood, and there are few strategies for preventing obesity-associated CRC [40].

Using liquid chromatography-tandem mass spectrometry (LC-MS/MS)-based metabolomics, our recent research identified sEH, a downstream enzyme involved in the CYP monooxygenase eicosanoid pathway [2], as a novel therapeutic target of obesity-induced colonic inflammation [41]. We found that sEH and its metabolic products (fatty acid diols) are overexpressed in the colon of obese mice. In addition, pharmacological inhibition or genetic ablation of sEH abolishes obesity-induced colonic inflammation (cytokine expression and immune cell infiltration into the colon) and the activation of pro-tumorigenic Wnt signaling (phosphorylation of GSK3β and expression of Axin2 in the colon) [41]. Together, these results demonstrate that sEH is required for obesity-induced colonic inflammation and activation of Wnt signaling, which are early events involved in the carcinogenesis of CRC and play critical roles in the initiation and promotion of CRC [42, 43]. These findings support that sEH could be a novel therapeutic target of obesity-associated CRC; sEH inhibitors, which are currently being evaluated in clinical trials targeting other disorders [10, 11], could be promising agents for preventing obesity-associated CRC.

### Conclusion

CRC is the third most common cancer, the second leading cause of cancer-related deaths in the United States, and a serious health problem in the United States. Furthermore, it is well established that obese individuals are at high-risk of developing CRC [38, 39], and obesity-associated CRC represents an unmet medical problem in the United States. Our recent research supports that CYP monooxygenases could be novel therapeutic targets of CRC [12], and sEH, a down-stream enzyme in the CYP eicosanoid pathway, could be a potential target of obesity-associated CRC [41]. A better understanding of the roles of CYP/sEH pathway in these diseases could help to develop novel strategies for prevention and/or treatment. Notably, it is of critical importance to test whether it is feasible to target sEH for preventing or treating obesity-associated CRC, since the pharmacological inhibitors of sEH are currently being evaluated in multiple human clinical trials targeting other human disorders [10, 11], and these drugs could be repurposed for preventing or treating obesity-associated CRC.
